# Quantification and Evaluation of the Role of Antielastin Autoantibodies in the Emphysematous Lung

**DOI:** 10.1155/2011/826160

**Published:** 2011-03-31

**Authors:** Teck Boon Low, Catherine M. Greene, Shane J. O'Neill, Noel G. McElvaney

**Affiliations:** Respiratory Research Division, Department of Medicine, Royal College of Surgeons in Ireland, Education and Research Centre, Beaumont Hospital, Dublin 9, Ireland

## Abstract

Chronic obstructive pulmonary disease (COPD) may be an autoimmune disease. Smoking causes an imbalance of proteases and antiproteases in the lung resulting in the generation of elastin peptides that can potentially act as autoantigens. Similar to COPD, Z alpha-1 antitrypsin deficiency (Z-A1ATD) and cystic fibrosis (CF) are associated with impaired pulmonary antiprotease defences leading to unopposed protease activity. Here, we show that there is a trend towards higher bronchoalveolar lavage fluid (BALF) antielastin antibody levels in COPD and Z-A1ATD and significantly lower levels in CF compared to control BALF; the lower levels in CF are due to the degradation of these antibodies by neutrophil elastase. We also provide evidence that these autoantibodies have the potential to induce T cell proliferation in the emphysematous lung. This study highlights that antielastin antibodies are tissue specific, can be detected at elevated levels in COPD and Z-A1ATD BALF despite their being no differences in their levels in plasma compared to controls, and suggests a therapeutic role for agents targeting these autoantibodies in the lungs.

## 1. Introduction

Chronic obstructive pulmonary disease (COPD) is characterized by poorly reversible airflow limitation that is usually progressive and associated with an abnormal inflammatory response of the lungs to noxious particles or gases [1]. Cigarette smoking is a well-known risk factor for developing COPD. Damage to extracellular matrix proteins, for example elastin, plays a major role in the pathology of COPD but also in other chronic inflammatory lung diseases such as Z alpha-1 antitrypsin deficiency (Z-A1ATD, a genetic form of emphysema) and cystic fibrosis. An imbalance of proteases and antiproteases in these chronically inflamed lungs can potentially generate neoantigens derived from elastin.

CD8^+^ and CD4^+^ T cells are abundant in the COPD lung [2, 3]. Cosio et al. [2] have suggested that in COPD, these cells may be activated by dendritic cells presenting unique antigens released during smoking-induced lung injury, for example, elastin peptides. The adaptive immune system recognises these antigens as foreign and triggers an immune reaction leading to the generation of autoantibodies.

In 2007 Lee et al. described the presence of antielastin autoantibodies in the plasma of individuals with COPD and showed that elastin peptides can induce proliferation of peripheral blood CD4+ T cells isolated from individuals with COPD but not control individuals nor asthma patients [4]. Choo later commented on this [5]; however, we [6] and others later disputed the singularity of this observation with respect to COPD by demonstrating that antielastin antibodies are also detectable, and present at even higher levels [7], in plasma of smoking controls. Cottin et al. also failed to detect the presence of circulating antielastin autoantibodies in combined pulmonary fibrosis and emphysema compared to controls [8]. 

The lack of systemic antielastin antibodies in COPD or other chronic inflammatory lung conditions does not preclude the possibility of local autoimmune responses in the lung playing an important role in disease pathogenesis; compartmentalised inflammatory responses are an inherent feature of inflammatory lung diseases. For example, Calabrese et al. demonstrated increased IL-32 expression in lung samples of COPD patients compared to controls [9], whereas systemic IL-32 levels were not found to be elevated in the plasma of a similar cohort of COPD patients [6]. 

In this study, we sought to detect the presence of antielastin autoantibodies in bronchoalveolar lavage fluid (BALF) from individuals with COPD, Z-A1AT deficiency and CF and compare levels to those in control BALF. We also aimed to determine a potential role for these antielastin antibodies in the emphysematous lung.

## 2. Materials and Methods

### 2.1. Study Population

A total of 45 subjects were included in this study—COPD (*N* = 14), Z-A1ATD (*N* = 5), cystic fibrosis (*N* = 15), and controls (*N* = 11). Study subjects were recruited from the respiratory clinics in Beaumont Hospital. All were diagnosed by standard criteria; individuals with CF were genotyped for CFTR mutations and had positive sweat testing of chloride >60 mmol/L; all individuals with Z-A1AT deficiency were homozygous for the Z allele and had serum A1AT <11 *μ*M; individuals with COPD had obstructive lung disease and a history of smoking. The majority of Z-A1AT deficiency and COPD study subjects had computed tomography evidence of emphysema. Individuals with known autoimmune diseases (e.g., connective tissue disorder, Graves disease), less than 18 years of age, or refusal to give consent were excluded from the study. None of the study subjects were on systemic corticosteroids. The control subjects were recruited from a nonpaid group of patients who were nonsmokers and were attending our respiratory outpatient unit for investigation of haemoptysis or chronic cough. Smoking and second-hand smoke exposure were excluded by history alone. All participants gave written, informed consent to participate in the study, which was approved by Beaumont Hospital Ethics Committee.

### 2.2. Pulmonary Function Testing

Pulmonary function tests of study subjects were measured using a spirometer using the acceptability standards outlined by the American Thoracic Society (ATS)/European Respiratory Society (ERS). Pulmonary function tests were performed three times in each subject with an acceptable technique. The predicted values of forced expiratory volume in one second (FEV1) are calculated according to the patient's age, height, gender, and ethnicity utilizing consistent reference values.

### 2.3. Bronchoscopy

Following informed consent using a protocol approved by Beaumont Hospital Ethics committee subjects underwent bronchoalveolar lavage (BAL) via a flexible fibreoptic bronchoscope. The upper respiratory tract was anaesthetized with 2% lignocaine. Supplemental oxygen was given throughout the procedure as a routine, and the oxygen saturation was monitored by continuous pulse oxymeter. Sixty mls of normal saline were instilled and suctioned back through the bronchoscope. The dilution factor of BAL fluid (BALF) was corrected by measuring total protein via Bradford assay.

### 2.4. Antielastin ELISA

Antielastin IgG antibodies in BALF were quantified by using a modified ELISA as described previously [6]. Assays were performed in duplicate. Anti-IgM, anti-IgA, anti-IgD, and anti-IgE antibodies were detected in a similar manner using appropriate secondary antibodies.

### 2.5. Neutrophil Elastase (NE) Activity Assay

NE activity was quantified in BALF using the NE substrate 3 mM N-methocysuccinyl-Ala-Ala-Pro-Val-p-nitroanilide (Sigma Aldrich). The changes in absorbance at 405 nm were recorded at 1-minute interval for 5 minutes and compared with an NE standard (Elastin Products Company) of known activity.

### 2.6. Cell Culture

Human lymphoblastic Jurkat E6.1 T cells (European Collection of Animal Cell Cultures, Salisbury, U.K.) were cultured in RPMI 1640 containing 10% FCS, 1% penicillin and streptomycin (Sigma-Aldrich) and were maintained at 37°C in a humidified atmosphere of 5% CO_2_. Jurkat T cells (1 × 10^5^) were cultured in 24-well plates onto which anti-CD3 mAb (R&D systems) had been immobilized. Wells were coated for 3 h at 37°C with 300 *μ*L PBS containing 5 *μ*g/mL anti-CD3 and washed three times with PBS before use. Cells were treated with BALF that contained antielastin or without antielastin antibodies. IL-12 (10 ng/mL) and IL-18 (30 ng/mL) (R&D systems) were used as a positive control.

### 2.7. Antielastin Antibody Depletion from BALF

Isopropanol was removed from a 1 mL HiTrap NHS-activated HP resin (GE Healthcare) column with 1 mM HCl prior to ligand coupling. Coupling with 0.5 mg/mL of elastin peptide (Elastin Product Company) was carried out for 30 minutes. Washing and deactivation of nonspecifically bound ligand was carried out as per the manufacturer's instructions. After further washing, antielastin antibodies were depleted from BALF by passing it through the elastin-linked column. The eluate was retained for Jurkat cell stimulation studies.

### 2.8. Cell Proliferation Assay

Cell proliferation assays were performed by adding 20 *μ*L of CellTiter 96 Aqueous One Solution Proliferation Assay in a humidified, 5% CO_2_ atmosphere for 4 hours (Promega, Madison, WI) to culture wells that contained anti-CD3 Jurkat T cells treated or not for 24 h with BALF. IL-12 (10 ng/mL) and IL-18 (30 ng/mL) (R&D systems) were used as a positive control. Absorbance at 490 nm was then measured using a 96-well microplate reader.

### 2.9. Statistics

Data are expressed as mean ± SD or ± SEM, as indicated. Test of normality was performed by the Kolmogorov-Smirnov test. All data were of nonparametrical distribution. Differences between two individual groups or more than two groups were assessed by *t*-test or ANOVA as appropriate. Statistical tests were performed using SPSS 15.0 and Prism 4.0 software. *P* values  <.05 were considered to be significant.

## 3. Results

### 3.1. Study Population Characteristics

A total of 45 individuals were included in this study (control, *n* = 11; COPD, *n* = 14; Z-A1AT deficiency, *n* = 5; CF, *n* = 15).  Table 1 provides details of their baseline characteristics, and Table 2 provides the severity of disease in COPD, Z-A1AT deficiency and CF based on the predicted percentage of FEV1.

### 3.2. Bronchoalveolar Lavage Fluid (BALF) Antielastin Autoantibodies (IgG, IgM, IgA, IgD, and IgE)

**Figure 1:**
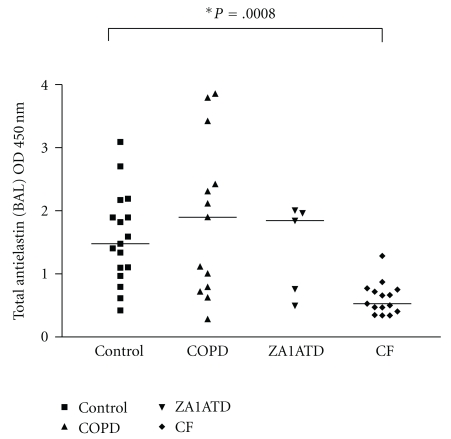
Total antielastin antibodies in bronchoalveolar lavage fluids were quantified in control (*N* = 11), COPD (*N* = 14), Z-A1ATD (*N* = 5) and CF (*N* = 15) samples by ELISA.

BALF levels of antielastin autoantibodies were quantified in individuals with COPD, Z-A1AT deficiency or CF and compared to controls. The results are shown in Figure 1. There is a trend towards higher BALF antielastin antibodies in Z-A1AT deficiency and COPD compared to controls. There is a significant reduction in BALF antielastin antibodies in CF compared to controls (*P* = .0008).

### 3.3. Neutrophil Elastase (NE) Activity

**Figure 2:**
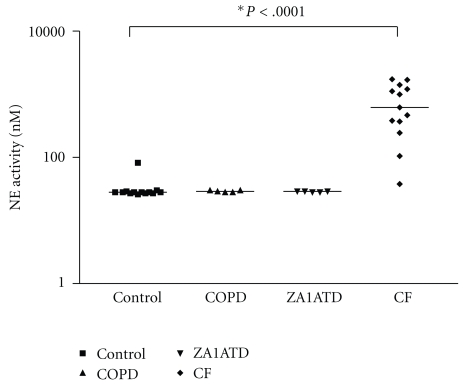
Neutrophil elastase activity. Neutrophil elastase activities were measured in bronchoalveolar lavage fluids of controls (*N* = 8) or patients with COPD (*N* = 5), Z-A1ATD (*N* = 5) or CF (*N* = 13) using N-methocysuccinyl-Ala-Ala-Pro-Val-p-nitroanilide.

We speculated that NE may be the factor responsible for the significantly lower BALF antielastin antibodies in the CF group. NE activities were measured in all 4 groups as shown in Figure 2. Similar to control BALF the COPD and Z-A1ATD samples did not have detectable NE activity. Free NE was detectable in CF BALF and levels were significantly higher than controls (*P* < .0001).

### 3.4. Neutrophil Elastase Degrades Antielastin Autoantibodies

**Figure 3:**
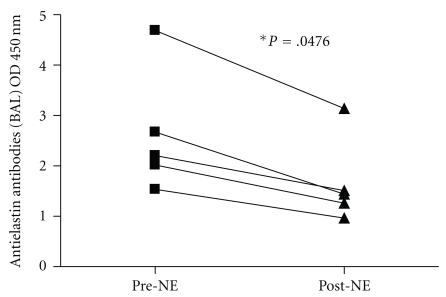
Antielastin antibodies levels pre- and post-NE. Bronchoalveolar lavage fluid antielastin antibodies levels were measured from 5 non-CF individuals pre- and post-NE treatment by ELISA where 1 *μ*M NE was added to 100 *μ*L BALF samples for 24 hours.

In order to confirm that the high burden of NE in the CF BALF is responsible for degrading BALF antielastin antibodies, five non-CF BALF samples with high antielastin antibodies levels were left untreated or treated with NE for 24 hours and antielastin antibody levels were quantified pre- and after treatment.  Figure 3 shows that there is a significant reduction in BALF antielastin antibodies post-NE treatment (*P* = .0476) confirming that NE can degrade BALF antielastin antibodies and suggesting that in the CF lung excessive levels of NE are responsible for the low BALF antielastin antibodies evident in this group.

### 3.5. COPD and Z-A1ATD BALF Can Induce T Cell Proliferation

**Figure 4:**
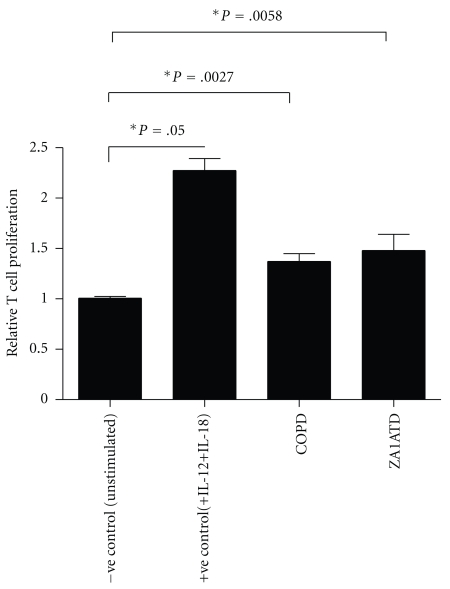
Effect of COPD and Z-A1AT BALF on T cell proliferation. T cell proliferation was measured in (1 × 10^5^) CD3-activated Jurkat T cells left untreated, stimulated with IL-12 (10 ng/mL) and IL-18 (30 ng/mL) or with Z-A1ATD BALF (500 *μ*L) or COPD BALF (500 *μ*L) for 24 hours at 37°C (*n* = 3).

Next, we assessed the potential functional effects of elevated antielastin antibodies in the COPD and Z-A1ATD lung. Given that COPD is known to be associated with increased intrapulmonary CD8^+^ T cell numbers, we investigated the effect of antielastin antibodies on T cell proliferation *in vitro*. CD3-activated Jurkat T cells were treated with BALF; costimulation with IL-12 and IL-18 was used as a positive control. Cell proliferation assays were performed, and the results are shown in Figure 4. Compared to control cells, IL-12/IL-18 stimulation induced T cell proliferation. Stimulation with COPD or Z-A1ATD BALF also caused T cell proliferation albeit less potently than the positive control.

### 3.6. Depletion of Antielastin Antibodies from BALF Decreases T Cell Proliferation

**Figure 5:**
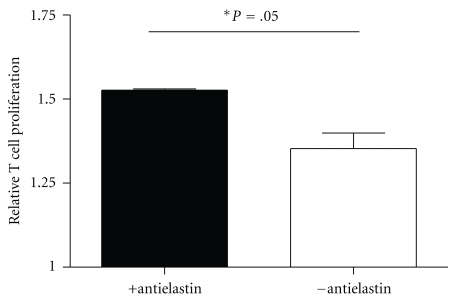
Effect of depletion of antielastin antibodies from Z-A1ATD BALF on T cell proliferation. T cell proliferation was measured in (1 × 10^5^) CD3-activated Jurkat T cells treated for 24 hours at 37°C with intact Z-A1ATD BALF (500 *μ*L) or BALF from which antielastin antibodies had been depleted (*n* = 3).

Using an elastin-conjugated resin column, we removed the antielastin antibodies from Z-A1ATD BALF and used the filtrates to stimulate T cell proliferation (Figure 5). Compared to complete BALF, the antielastin antibody-depleted BALF induced less T cell proliferation, (*P* = .05). This implicates antielastin antibodies as a factor contributing to the T cell proliferative properties of Z-A1AT BALF.

## 4. Discussion

A number of studies have suggested that COPD is likely to have an autoimmune component [4, 10, 11]. Although we have previously reported a lack of elevated systemic antielastin autoantibody levels in COPD or other chronic inflammatory lung disease patients that is Z-A1ATD and CF, here, we present data showing that antielastin antibody levels in bronchoalveolar lavage fluid tend to be higher in COPD and Z-A1ATD compared to controls. Although the results are not significant the number of patients in each group is small. Nonetheless our findings support the concept that local intrapulmonary autoimmunity may contribute to disease pathogenesis in emphysema.

In an attempt to identify a functional role for the higher than normal levels of antielastin autoantibodies in the COPD and Z-A1ATD lung, we focussed on their possible impact on T cell proliferation. We did this because COPD is associated with higher than normal intrapulmonary numbers of T cells [12–14]. Our investigations revealed that COPD and Z-A1ATD BALF could induce proliferation of CD3-activated Jurkat T cells. It is already known that IL-18 is elevated in COPD BALF [15], and this likely contributes to the enhanced T cell proliferation observed in cells stimulated with BALF. However, our data clearly implicates antielastin antibodies as a further factor in BALF potentially responsible for T cell proliferation. Specific depletion of these antibodies from BALF significantly decreased the T cell proliferative effect of Z-A1ATD BALF. Additional experiments such as spiking of BALF with purified antielastin antibodies or additional T cell functional studies quantifying cytokine expression were unfortunately not possible here due to sample limitation.

A secondary finding of this work was the interesting observation that antielastin antibody levels are significantly lower in the CF lung compared to healthy controls and that the serine protease neutrophil elastase may be responsible for degradation of these autoantibodies *in vivo*. The CF lung is a milieu with a high-protease burden [16–18]; endogenous and pathogen-derived proteases contribute significantly to a dysfunctional innate immune response [19]. NE, the most abundant serine protease in the CF lung, can cleave a wide range of substrates including cell surface receptors, extracellular matrix proteins, cytokines, antiproteases, defensins, and immunoglobulins [19, 20]. For example, NE can degrade IgM rheumatoid factor [21, 22]. Our data shows that antielastin antibodies represent a new subclass of immunoglobulins that can be degraded by NE. Although we did not evaluate the effect of other pulmonary proteases on antielastin antibody degradation in this study, it would be interesting to know whether other classes of proteases such as cysteine or metalloproteases induce a similar effect. 

Interestingly T cell infiltration is not a major feature of CF, and the lack of antielastin autoantibodies in the CF lung represents a good corollary to COPD, where both increased numbers of T cells and higher than normal antielastin antibodies have been detected. Ongoing studies will determine T cells number *in vivo* in the Z-A1ATD lung. During acute exacerbations in COPD or Z-A1ATD patients, NE levels rise sharply possibly leading to antielastin autoantibody degradation that may impact on T cell proliferation. Further studies to determine whether targeting antielastin autoantibodies could have therapeutic implications for patients with COPD and Z-A1ATD are warranted.

## 5. Conclusion

In conclusion, our findings show that BALF antielastin autoantibodies are present in COPD and Z-A1ATD but not in CF, and these antibodies can cause T cell proliferation. The lack of antielastin antibodies in CF is due to NE in degradation.

## Figures and Tables

**Table 1:** Patient characteristics.

	Control	COPD	Z-A1ATD	CF
No. of subjects	11	14	5	15
Age, yr (±SD)*¹*	59.3 ± 24.1	67.1 ± 8.9	41.6 ± 19.8	28.3 ± 10.2
Sex, % M/F²	73/27	64/36	60/40	53/47
BMI³, kg/m² (±SD)*¹*	25.6 ± 1.7	24.8 ± 5.3	27.3 ± 3.2	24.1 ± 2.3
FEV1^4^, % predicted (±SD)*¹*	—	45.6 ± 23.0	92.0 ± 25.7	43.5 ± 26.4
FVC^5^, % predicted (±SD)*¹*	—	74.4 ± 29.2	106.6 ± 16.5	62.1 ± 23.4

*¹*±SD, ±standard deviation.

²M/F, male/female.

³BMI, body mass index.

^4^FEV1, forced expiratory volume in 1 second.

^5^FVC, forced vital capacity.

**Table 2:** The severity of disease in COPD, Z-A1ATD, and CF based on the predicted percentage of FEV1.

	COPD	Z-A1ATD	CF
FEV1 (>80% predicted)	14%	40%	6%
FEV1 (50–80% predicted)	21%	60%	27%
FEV1 (30–50% predicted)	36%	—	27%
FEV1 (<30% predicted)	29%	—	40%
